# β-Glucan Size Controls Dectin-1-Mediated Immune Responses in Human Dendritic Cells by Regulating IL-1β Production

**DOI:** 10.3389/fimmu.2017.00791

**Published:** 2017-07-07

**Authors:** Matthew J. Elder, Steve J. Webster, Ronnie Chee, David L. Williams, J. S. Hill Gaston, Jane C. Goodall

**Affiliations:** ^1^Department of Medicine, Addenbrooke’s Hospital, University of Cambridge, Cambridge, United Kingdom; ^2^Department of Immunology, Royal Free Hospital, London, United Kingdom; ^3^Department of Surgery and Center for Inflammation, Infectious Disease and Immunity, James H. Quillen College of Medicine, East Tennessee State University, Johnson City, TN, United States

**Keywords:** dectin-1, β-glucan, IL-1β, dendritic cell, reactive oxygen species, phagocytosis

## Abstract

Dectin-1/CLEC7A is a pattern recognition receptor that recognizes β-1,3 glucans, and its stimulation initiates signaling events characterized by the production of inflammatory cytokines from human dendritic cells (DCs) required for antifungal immunity. β-glucans differ greatly in size, structure, and ability to activate effector immune responses from DC; as such, small particulate β-glucans are thought to be poor activators of innate immunity. We show that β-glucan particle size is a critical factor contributing to the secretion of cytokines from human DC; large β-glucan-stimulated DC generate significantly more IL-1β, IL-6, and IL-23 compared to those stimulated with the smaller β-glucans. In marked contrast, the secretion of TSLP and CCL22 were found to be insensitive to β-glucan particle size. Furthermore, we show that the capacity to induce phagocytosis, and the relative IL-1β production determined by β-glucan size, regulates the composition of the cytokine milieu generated from DC. This suggests that β-glucan particle size is critically important in orchestrating the nature of the immune response to fungi.

## Introduction

Pattern recognition receptor (PRR) signaling pathways are a critical component of innate host defense against infectious pathogens. Human dectin-1/CLEC7A is a PRR of the C-type lectin family of receptors, predominantly expressed on myeloid-derived cells (1, 2), and recognizes exposed β-1,3 glucans (β-glucans) present on fungi and bacteria cell walls (3). Engagement of dectin-1 stimulates activation of its unconventional immunoreceptor tyrosine activation motif (hemITAM) (3–6), followed by subsequent activation of downstream Syk-dependent and -independent signaling events (5, 7, 8). The engagement of dectin-1 signaling has been reported to initiate a plethora of immunological defense mechanisms including phagocytosis (2, 9, 10), reactive oxygen species (ROS) production (6, 8), inflammasome complex activation (8, 11–13), and the release of inflammatory cytokines (7, 14–16), required for antifungal immunity.

β-Glucan molecules exhibit heterogeneity in size, structure, and capacity to activate immunological effector responses (10, 14, 15, 17, 18). Functional dectin-1 signaling requires the β-glucan to be particulate, or in an immobilized form (18), and the size and structure of the β-glucan particulate is key to dictating the magnitude of the inflammatory immune response initiated by dendritic cells (DCs) in response to stimulation (14, 15, 17). It has been suggested that β-glucan size controls the capacity of myeloid-derived cells to phagocytose fungal products. Therefore, dectin-1-mediated phagocytosis is thought to attenuate the production of inflammatory cytokines from DC (14, 15), with small particulate β-glucans deemed poor activators of innate immunity. Furthermore, fungal species such as *Candida albicans* (*C. albicans*) exist as both colonizing yeast and invasive hyphae, and structural differences exist in β-glucans derived from these differing forms—hyphae exhibit a cyclical β-glucan structure while yeast β-glucans are linear (17). These structural differences have been shown to modulate the production of the inflammatory cytokine IL-1β (17) and alter the rate of phagocytosis (10), with small β-glucan particulates inducing rapid phagocytosis and limited IL-1β. It is well established that the production of IL-1β along with IL-6 and IL-23 are required for antifungal immunity in part by priming the adaptive immune response to induce the differentiation of CD4^+^ T cells to T_H_1/T_H_17 cells (3, 12, 16, 19). Therefore, the aim of this study was to further investigate how β-glucan size controls dectin-1-mediated responses in primary human monocyte-derived dendritic cells (mDCs). Patients with chronic granulomatous disease (CGD) are unable to generate nicotinamide adenine dinucleotide phosphate-oxidase (NADPH)-derived ROS. As a result, they have reduced antifungal killing activity and suffer from recurrent fungal infections (20–22). Therefore, a better understanding of how β-glucan size regulates key antifungal mechanisms, including the need for ROS, could lead to improved management of infection.

We report here that the size of β-glucan particles regulates the production of certain cytokines (IL-1β, IL-6, and IL-23), while others (TSLP and CCL22) were unaffected by β-glucan size. Furthermore, we show that the regulation of IL-1β production and phagocytosis, both determined by β-glucan size, provides a key molecular checkpoint in eliciting appropriate cytokine secretion from mDC in response to dectin-1 activation.

## Results

### β-Glucan Particle Size Differentially Affects Cytokine Secretion in Human mDC

**Figure 1:**
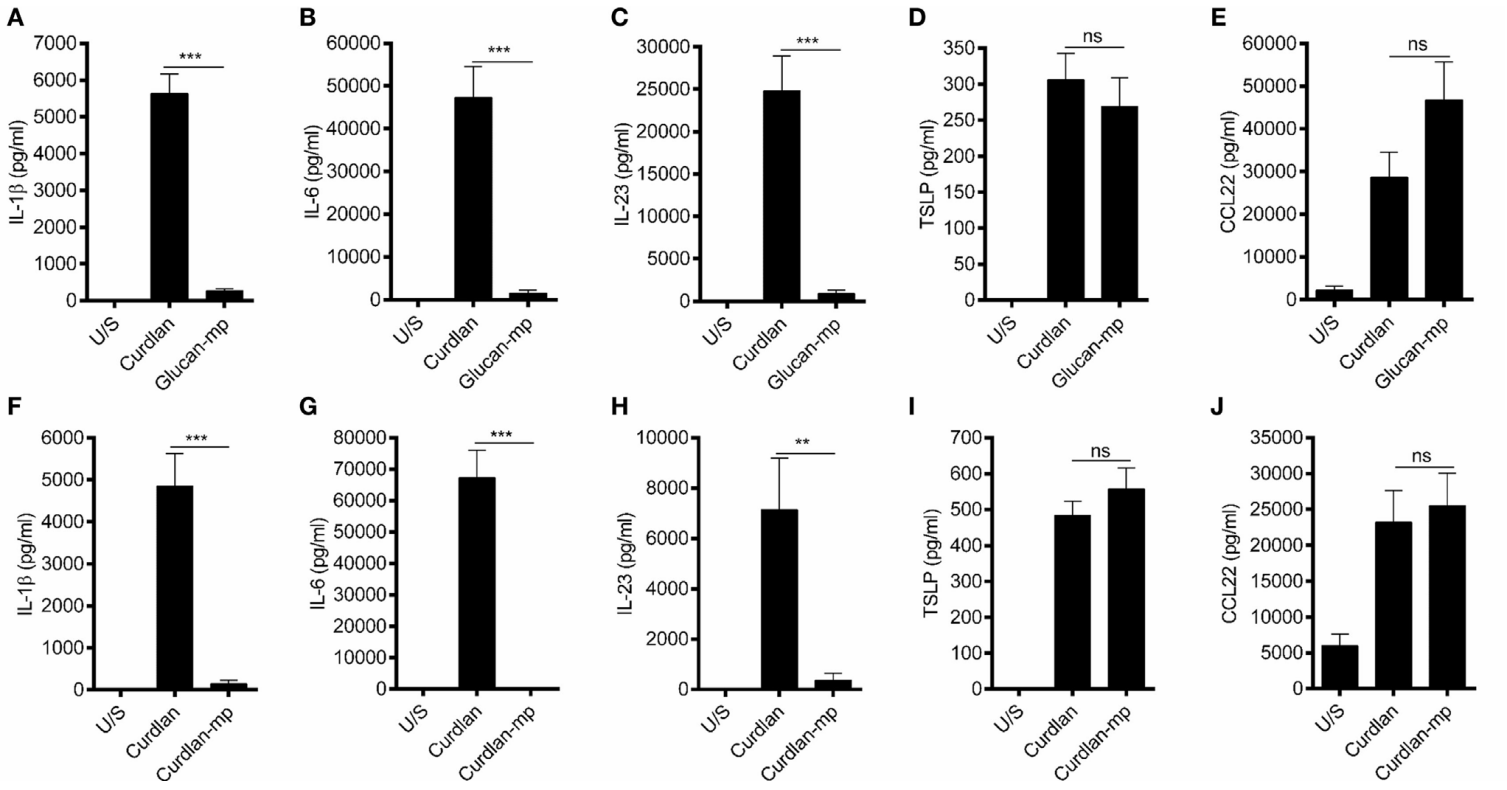
β-Glucan particle size differentially affects cytokine secretion by human monocyte-derived dendritic cell (mDC). **(A–E)** Human mDCs were stimulated with curdlan or β-glucan-microparticles (glucan-mp) for 24 h (*n* = 8 donors), and with curdlan or curdlan-microparticles (curdlan-mp) (*n* = 7 donors) **(F–J)** and IL-1β, IL-6, IL-23, TSLP, and CCL22 measured by enzyme-linked immunosorbent assay. Cumulative data displayed as mean ±SEM.

**Figure 2:**
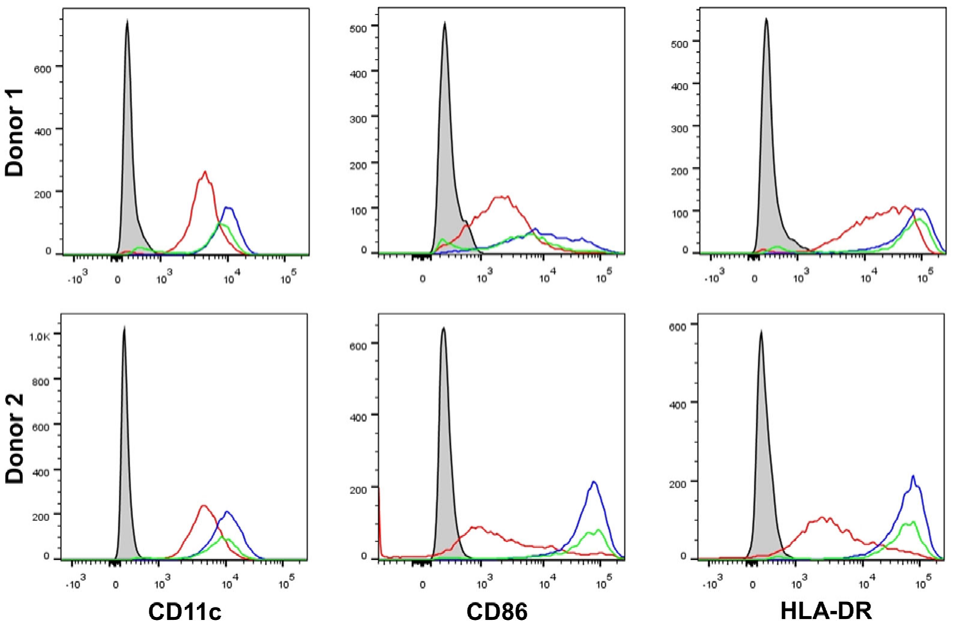
β-Glucan particle size does not affect activation status of human monocyte-derived dendritic cell (mDC). Human mDCs were stimulated with either curdlan or glucan-mp for 24 h and CD11c, CD86, and HLA-DR expression measured to determine mDC activation by flow cytometry (*n* = 2 donors). Gray-filled profile represents unstimulated unstained mDC, the red line represents unstimulated stained mDC, the blue line represents curdlan-stimulated stained mDC, the green line represents glucan-mp-stimulated stained mDC.

**Figure 3:**
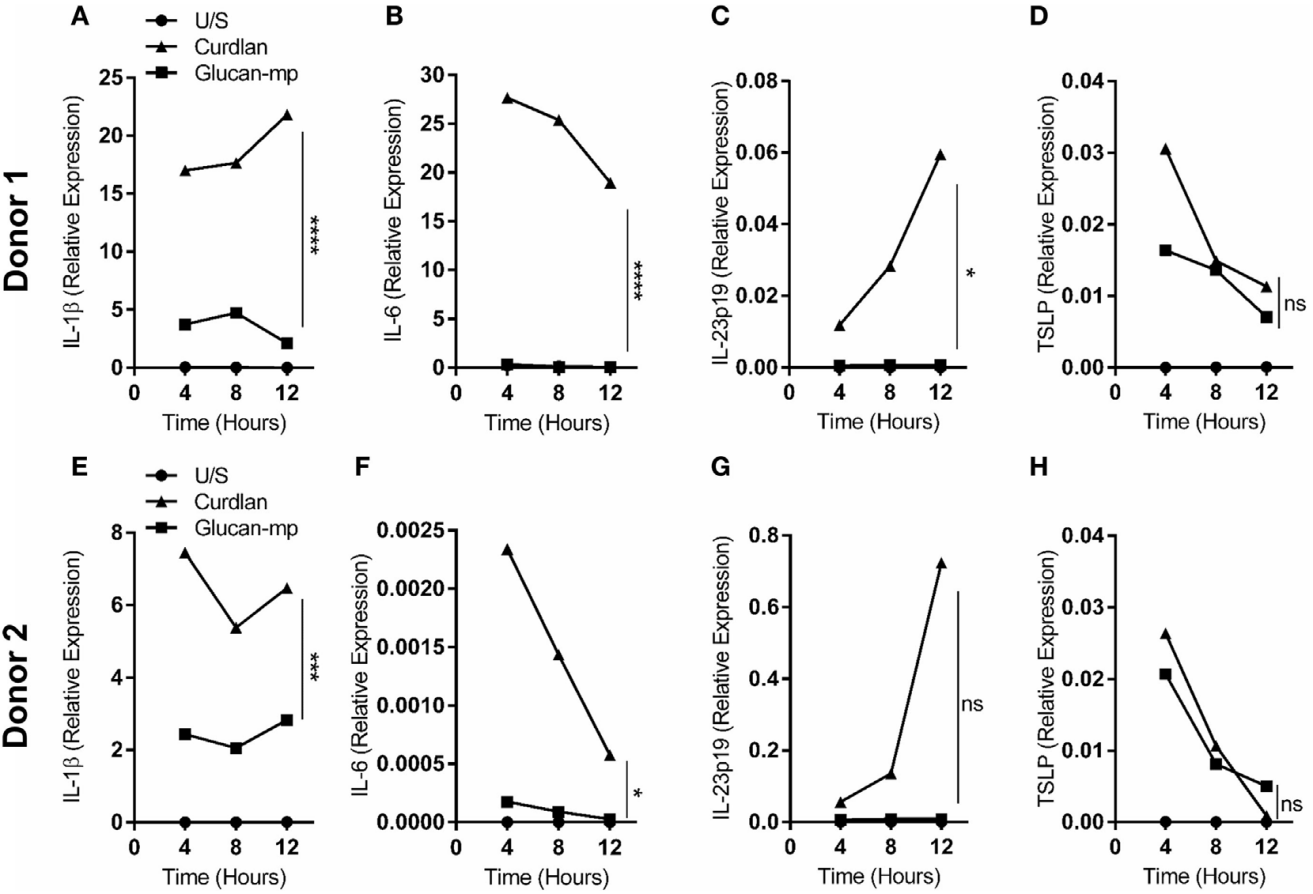
IL-1β, IL-6, IL-23p19, and TSLP mRNA expression by monocyte-derived dendritic cell (mDC) in response to curdlan or β-glucan-microparticles reflects cytokine production. **(A–H)** Human mDCs were stimulated with either curdlan or glucan-mp for 4, 8, or 12 h (*n* = 2 donors). IL-1β, IL-6, IL-23p19, and TSLP mRNA expression were measured by quantitative real-time polymerase chain reaction as relative expression (2^−ΔCt^), using HPRT expression as a housekeeping gene. Data points presented as the mean of replicate values.

It has been previously shown that β-glucan particle size significantly affects inflammatory cytokine secretion from myeloid-derived cells with smaller particulate forms of β-glucan found to be weak inducers of cytokine responses. This study aimed to characterize further the differences in cytokine responses elicited by either the large particulate β-glucan, curdlan (up to 0.2 mm diameter), or β-glucan-microparticles (glucan-mp; 1–5 µm diameter) (14), in human mDC by measuring IL-1β, IL-6, IL-23, TSLP, and CCL22 secretion in response to these different stimuli. Characterization of both curdlan and glucan-mp by high field 1H-NMR showed these β-glucans to have very similar primary structures (Supplementary Image 1). mDC stimulated with curdlan produced large amounts of IL-1β, IL-6, and IL-23, as compared to glucan-mp stimulated mDC secreted much reduced, though still detectable, quantities of IL-1β, IL-6, and IL-23 (Figures 1A–C). In marked contrast, curdlan- and glucan-mp induced secretion of similar quantities of TSLP and CCL22 (Figures 1D,E). However, since curdlan and the glucan-mp were derived from different microorganisms, *A. faecalis* (a Gram-negative bacterium) and the yeast *S. cerevisiae*, respectively, we wished to ensure that the findings were not due to differences in the species from which they were prepared. Therefore, we produced curdlan microparticles (curdlan-mp), using a previously described sonication protocol (14). Once more, we observed much reduced amounts of IL-1β, IL-6, and IL-23 in response to curdlan-mp as compared to curdlan, but no difference in TSLP or CCL22 (Figures 1F–J). Additionally, mDC stimulated with curdlan-or glucan-mp displayed no difference in activation status, as similar levels of surface expression of CD11c, CD86, and HLA-DR were observed (Figure 2). Thus, the results were consistent with a difference in the size of the different β-glucan preparations affecting cytokine production. To examine whether the effects observed were due to differences in gene transcription, we also tested whether particle size resulted in similar differences in cytokine gene transcription and found that IL-1β, IL-6, IL-23p19, and TSLP mRNA expression mirrored the data on secretion of each cytokine (Figures 3A–H). Thus, small particles induce reduced inflammatory gene transcription compared to larger particles.

### The Quantity of IL-1β Stimulated by a β-Glucan Particle Is a Key Factor That Determines the Secretion of Other Cytokines by mDC

**Figure 4:**
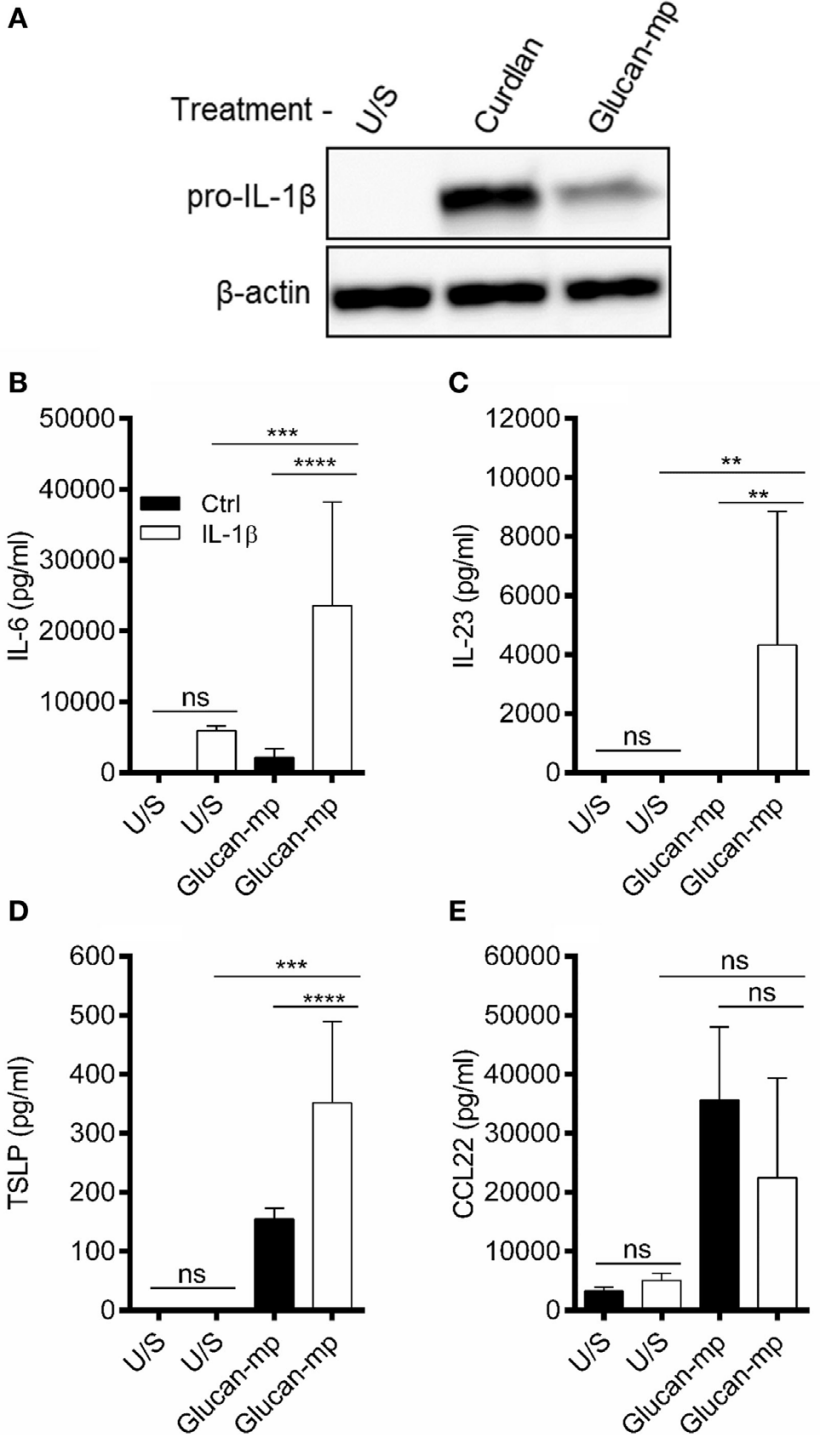
IL-1β concentration regulates glucan-mp-induced IL-6, IL-23, and TSLP secretion in monocyte-derived dendritic cell (mDC). **(A)** Human mDCs were stimulated with curdlan or glucan-mp for 8 h and pro-IL-1β protein expression measured by immunoblot (representative experiment presented; one of three). **(B–E)** Human mDC stimulated with glucan-mp and recombinant IL-1β simultaneously for 24 h (*n* = 6 donors). IL-6, IL-23, TSLP, and CCL22 were measured by enzyme-linked immunosorbent assay, with cumulative data displayed as mean ± SEM.

Our data suggested that small particulate β-glucans are not invariably poor inducers of cytokine secretion, but that certain cytokines are more sensitive to the size of the β-glucan particle used. Previous work has highlighted the importance of IL-1β secretion in orchestrating dectin-1-mediated immune responses (12, 16, 23). Therefore, we examined whether the amount of IL-1β stimulated by β-glucan particles of different sizes is critical in determining the amounts of other cytokines produced. We showed that pro-IL-1β protein expression in response to glucan-mp-stimulation was markedly reduced compared to curdlan stimulation (Figure 4A). Furthermore, exogenous recombinant IL-1β added at similar concentrations to that produced by curdlan stimulation recovered glucan-mp IL-6 and IL-23 secretion to levels equivalent to those produced with curdlan stimulation (Figures 4B,C). In contrast, amounts of CCL22 secreted were unaffected by addition of exogenous IL-1β (Figure 4E), while TSLP secretion was augmented, but only approximately twofold (Figure 4D), as compared to the >10-fold increase seen for IL-6 and IL-23. This result is in agreement with our previous finding that there is a requirement for signaling through the IL-1 receptor for optimal TSLP production (23). However, it is clear that unlike with IL-6 and IL-23, the additional IL-1β generated with curdlan stimulation of mDC is not essential for TSLP expression as the relatively low amounts of IL-1β produced in response to glucan-mp are sufficient for substantial secretion of TSLP.

### NADPH Oxidase-Derived ROS Are Required for IL-1β Secretion from mDC Stimulated with Glucan-mp, but Not with Curdlan

**Figure 5:**
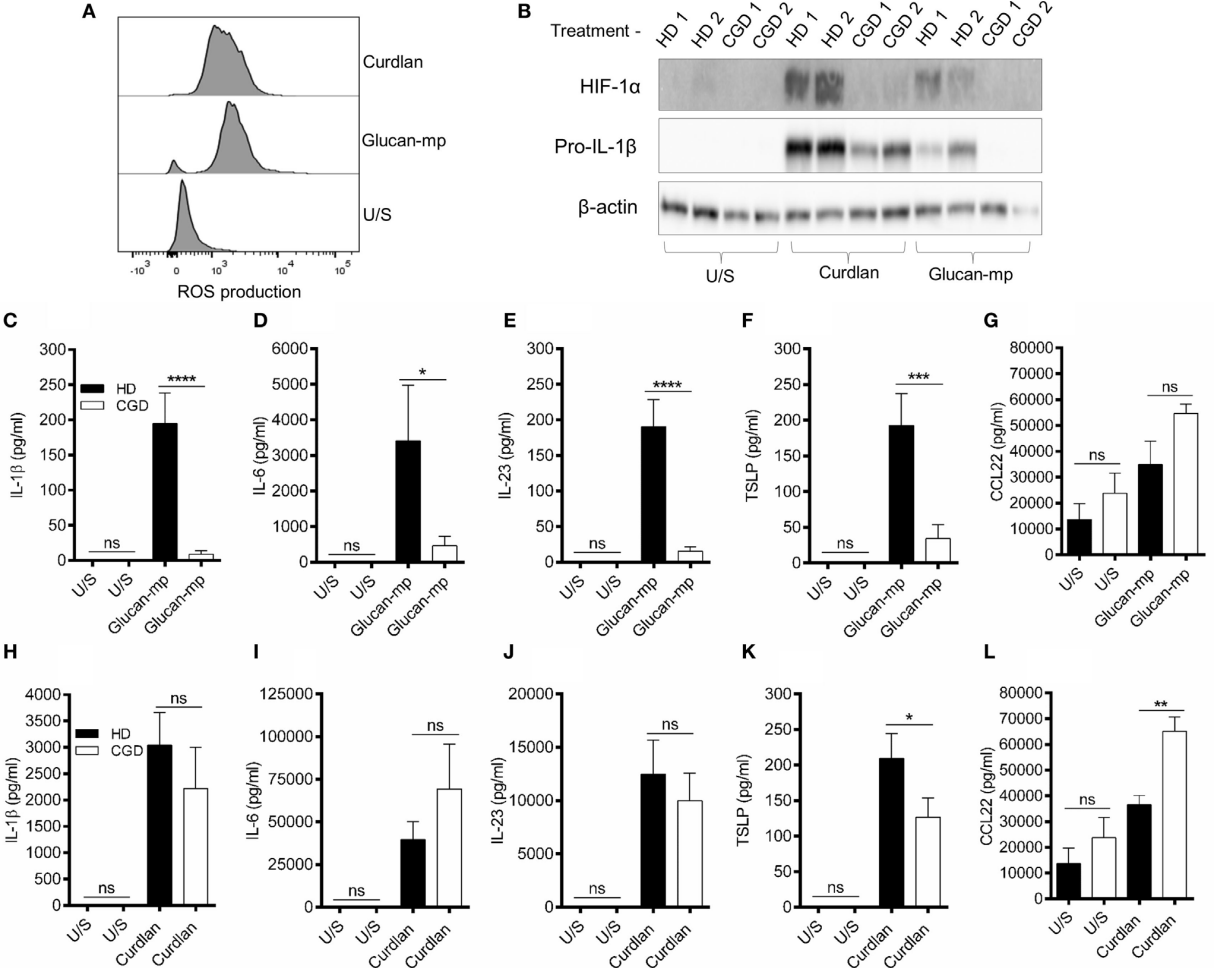
β-Glucan size affects the requirement for reactive oxygen species (ROS) in IL-1β induction. **(A)** Human monocyte-derived dendritic cell (mDC) stimulated with curdlan or glucan-mp for 8 h. ROS were detected by incubating cells with CellRox Green fluorescence dye and analysis by flow cytometry (representative experiment of two) presented, **(B)** Human mDCs from healthy donors or chronic granulomatous disease (CGD) patients were stimulated with curdlan or glucan-mp for 8 h. HIF-1α and pro-IL-1β protein expression measured by immunoblot (*n* = 2 donors). **(C–L)** Human mDC from healthy donors or CGD patients were stimulated with curdlan or glucan-mp for 24 h (*n* = 6 donors). IL-1β, IL-6, IL-23, TSLP, and CCL22 secretion were measured by enzyme-linked immunosorbent assay, with cumulative data displayed as mean ± SEM.

Since the amounts of IL-1β produced by mDC in response to large and small β-glucan particles differed substantially, we wished to examine the signaling requirements for the IL-1β production by each form of β-glucan. There are numerous reports showing the importance of generation of ROS, and the induction of hypoxia-inducible factor 1-alpha (HIF-1α), to induce the changes in cellular metabolism required for IL-1β expression during stimulation through dectin-1 (6, 8, 24, 25). We showed that curdlan and glucan-mp induced similar quantities of ROS in mDC (Figure 5A). They also both induced HIF-1α expression, though this was more marked with curdlan (Figure 5B). To determine the contribution of NADPH-derived ROS (termed ROS from this point) to HIF-1α expression and the induction of IL-1β, we generated mDC from patients with CGD; these patients have mutations in genes encoding components of the NADPH oxidase complex and, therefore, cannot generate ROS. When mDC from CGD patients were stimulated with glucan-mp, neither HIF-1α, nor pro-IL-1β were detected (Figure 5B), indicating that both required NADPH oxidase activity and likely ROS production. In contrast, curdlan stimulation induced pro-IL-1β in mDC from CGD patients, although this was reduced compared to healthy controls, and HIF-1α was not detected, thus indicating a ROS/HIF-1α-independent mechanism of pro-IL-1β production. Accordingly, IL-1β, IL-6, IL-23, and TSLP secretion was effectively abolished in mDC from CGD patients responding to glucan-mp, but not significantly affected when the same cells responded to curdlan (Figures 5C–L). As expected from the findings reported above, normal levels of IL-6 (Figure 5I), IL-23 (Figure 5J), and near normal levels of TSLP (Figure 5K) were produced by mDC from CGD patients responding to curdlan, since sufficient IL-1β was produced even in the absence of ROS and HIF-1α induction. Both curdlan and glucan-mp-induced CCL22 were insensitive to ROS production (Figures 5G,L), with a suggestion of enhanced levels from CGD patients. Thus, these experiments showed that IL-1β production in response to curdlan proceeds in the absence of ROS (and of HIF-1α), whereas this does not occur when glucan-mp are used to stimulate mDC.

### Inhibition of β-Glucan Internalization Allows IL-1β to Be Produced and Only Partially Requires NADPH Oxidase-Derived ROS

**Figure 6:**
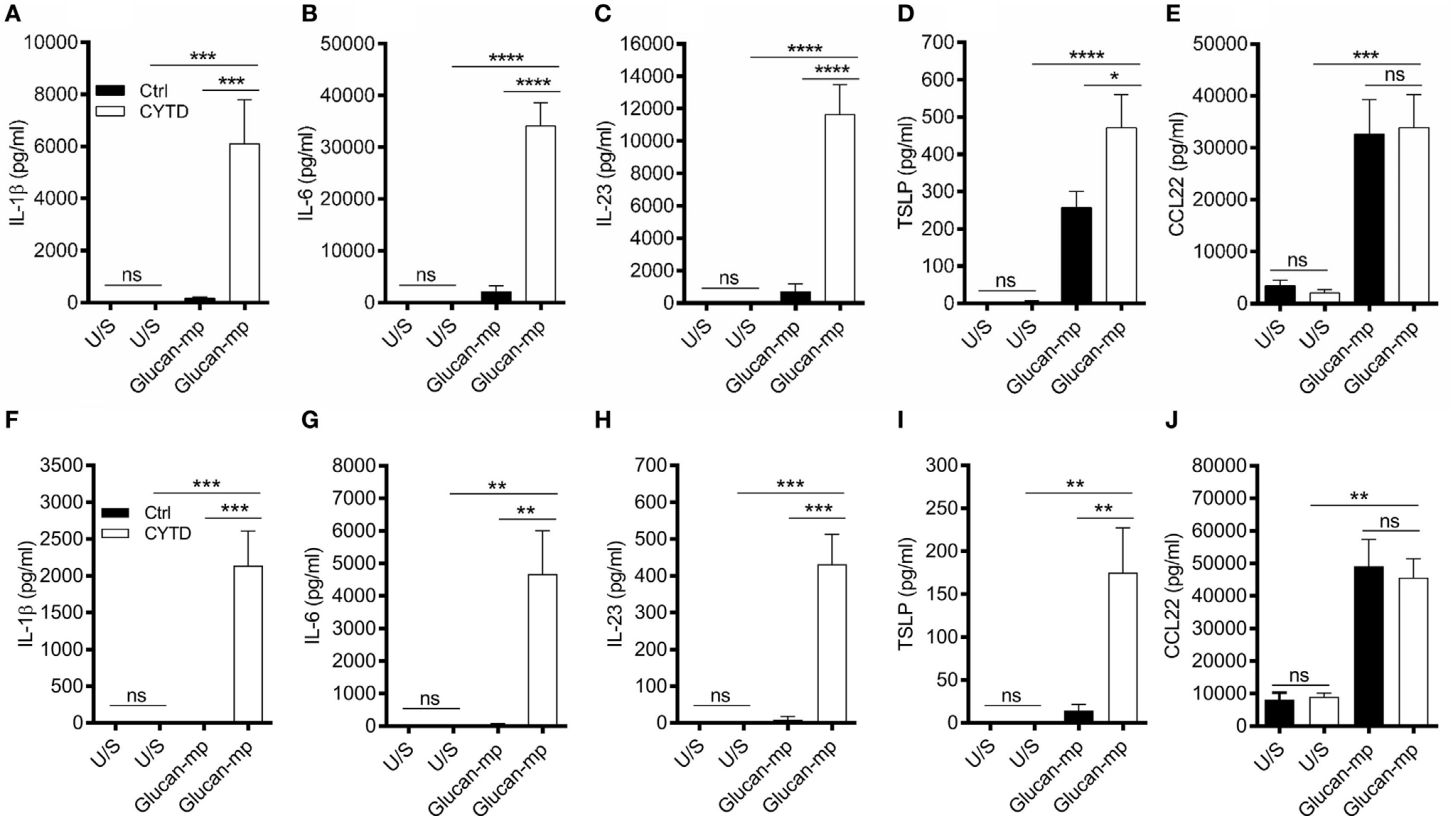
Treatment with cytochalasin D (CYTD) enhances IL-1β induction by monocyte-derived dendritic cell (mDC) from both healthy donors and chronic granulomatous disease (CGD) patients stimulated with glucan-mp. **(A–E)** Human mDCs were preincubated with CYTD for 1 h and then stimulated with glucan-mp for 24 h (*n* = 6 donors). **(F–J)** Human mDCs derived from CGD patients were preincubated with CYTD for 1 h and then stimulated with glucan-mp for 24 h (*n* = 3 donors). IL-1β, IL-6, IL-23, TSLP, and CCL22 were measured by enzyme-linked immunosorbent assay.

**Figure 7:**
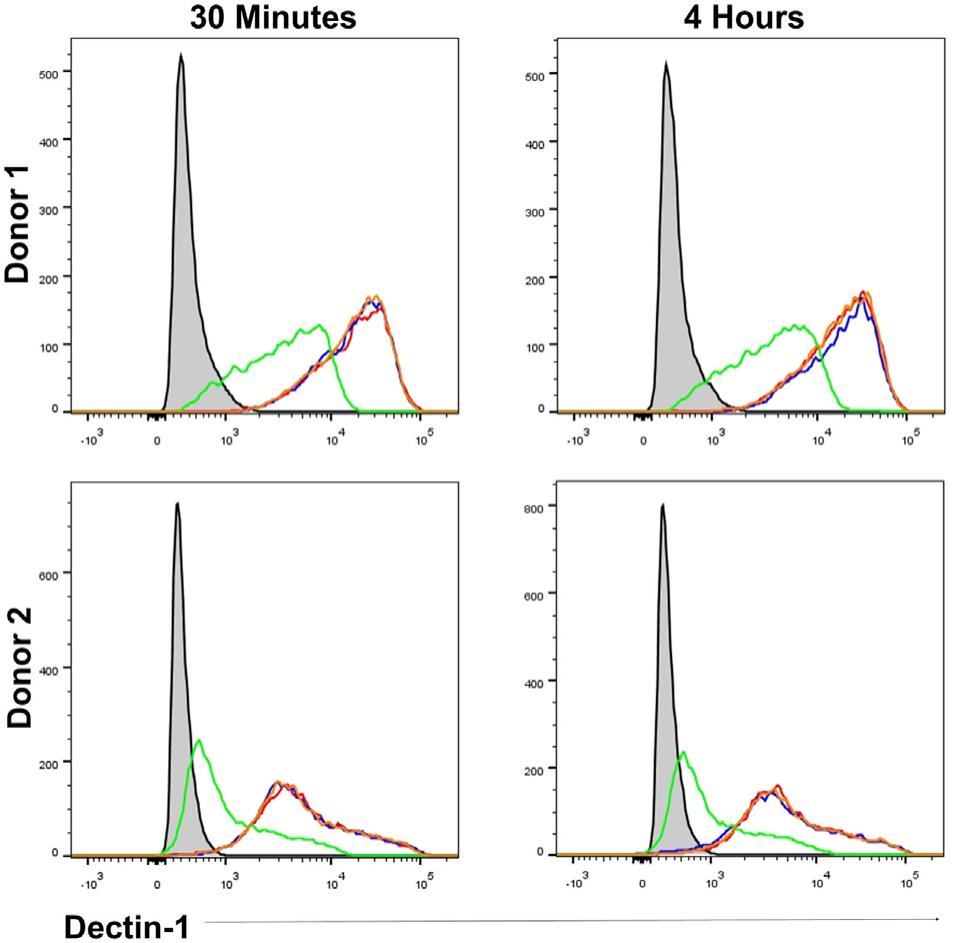
β-Glucan particle size affects surface dectin-1 expression on human monocyte-derived dendritic cell (mDC). Human mDC stimulated with either curdlan, glucan-mp, or glucan-mp subsequent to cytochalasin D (CYTD) pretreatment, for 30 min or 4 h (*n* = 2 donors). Dectin-1 expression following stimulation was determined relative to unstimulated stained mDC by flow cytometry. Gray-filled profile represents unstimulated unstained mDC, the red line represents unstimulated stained mDC, the blue line represents curdlan stimulated stained mDC, the green line represents glucan-mp stimulated stained mDC, and the orange line represents glucan-mp stimulated stained mDC subsequent to CYTD pretreatment (note that the red, blue, and orange lines essentially overlie each other).

As β-glucan size affected the production of IL-1β, and the requirement for NADPH oxidase activity to produce IL-1β, we investigated the mechanism accounting for the effects of particle size. The size of β-glucan particles affects the capacity of murine myeloid-derived cells to phagocytose them and induce inflammatory cytokines (14, 15). Thus, we treated human mDC with cytochalasin D (CYTD) to prevent glucan-mp internalization by phagocytosis and found that this substantially increased the amounts of IL-1β, IL-6, and IL-23 produced in response to glucan-mp, to levels equivalent to those obtained with curdlan stimulation; there was a less marked but significant increase in TSLP production, but no effect on CCL22 secretion since this was already maximal with glucan-mp (Figures 6A–E). Therefore, the inhibition of phagocytosis of glucan-mp had the same effect as the supplementation of glucan-mp-stimulated mDC cultures with exogenous IL-1β. The enhancement of glucan-mp-induced cytokine production by CYTD was independent of NADPH oxidase activity and likely ROS production, since mDC from CGD patients also produced augmented amounts of these cytokines when treated with CYTD (Figures 6F–I). Lack of NADPH oxidase activity had no effect on CCL22 secretion (Figure 6J). To further confirm whether these differences were a result of dectin-1-mediated phagocytosis, we examined the relative capacity of curdlan and glucan-mp to induce a decrease in dectin-1 surface expression on mDC. Glucan-mp-stimulated mDC induced a rapid decrease in surface dectin-1 expression 30 min poststimulation, whereas expression on mDC during curdlan stimulation barely changed in comparison to unstimulated stained controls, and this expression was maintained 4 h post-curdlan stimulation (Figure 7). Moreover, CYTD pretreatment of mDC stimulated with glucan-mp inhibited dectin-1 internalization (Figure 7) and likely prolonged signaling through dectin-1.

**Figure 8:**
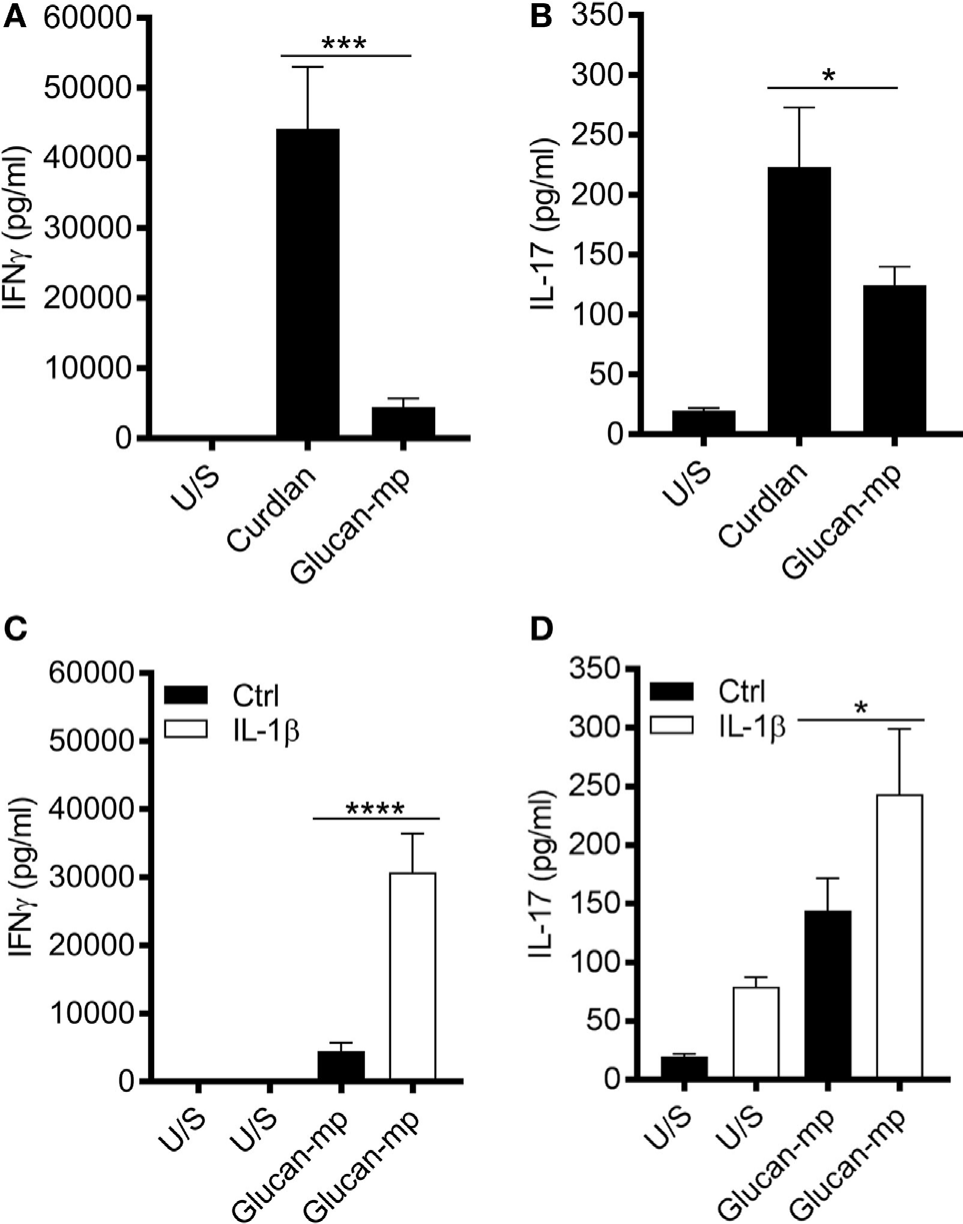
Naïve CD4^+^ T cells produce higher amounts of IL-17 and IFNγ when stimulated in conditioned media derived from curdlan-stimulated compared to glucan-mp-stimulated monocyte-derived dendritic cell (mDC). **(A–D)** CD3/CD28-stimulated naïve CD4^+^ T cells were cultured in supernatants derived from mDC prestimulated with either curdlan, glucan-mp, or glucan-mp supplemented with recombinant IL-1β simultaneously for 7 days (*n* = 4 donors). IL-17 and IFNγ measured by enzyme-linked immunosorbent assay. Cumulative data displayed as mean ± SEM.

It is well established that dectin-1 signaling promotes the differentiation of CD4^+^ T cells to T_H_1- and T_H_17-cells (3, 16, 26, 27); however, as β-glucan size significantly affected cytokine secretion from mDC, we investigated whether this had an impact on CD4^+^ T cell differentiation. CD3/CD28-stimulated naïve CD4^+^ T cells were cultured in supernatants derived from mDC prestimulated with either curdlan or glucan-mp. CD4^+^ T cells cultured in conditioned media derived from curdlan-stimulated mDC produced significantly higher levels of IL-17 and IFNγ compared to those CD4^+^ T cells cultured in conditioned media from glucan-mp stimulated mDC (Figures 8A,B). This increased production of IL-17 and IFNγ observed in curdlan-treated cells could be replicated when glucan-mp-stimulated mDCs were supplemented with recombinant IL-1β (Figures 8C,D).

Together these data show that failure to phagocytose β-glucan containing particles, as occurs with curdlan, or with glucan-mp in cells treated with CYTD, allows the production of IL-1β in a manner independent of NADPH oxidase activity. However, when phagocytosis of glucan-mp proceeds normally, the small amounts of IL-1β secreted requires NADPH activity, and is, therefore, regulated by an alternative mechanism compared to larger β-glucan particles that are not internalized. Therefore, the small amounts of IL-1β produced in response to internalized glucan-mp are insufficient to allow production of substantial amounts of IL-6 or IL-23 and this is the mechanism by which reduced inflammatory responses to glucan-mp occur. Furthermore, the differences in cytokine milieu generated as a result of β-glucan size modify the capacity to drive T_H_1- and T_H_17-cell differentiation.

## Discussion

To examine cytokine secretion induced in human mDC through dectin-1, we utilized β-glucans of differing size: curdlan (large particulate) and glucan-mp (small particulate). Similar to previous studies in mice (14, 15), we showed that curdlan-stimulated human mDC generated significantly more IL-1β, IL-6, and IL-23 compared to those stimulated with the smaller glucan-mp, and these results reflected particle size since curdlan prepared as a small particulate also produced much lower levels of these cytokines. In marked contrast, secretion of TSLP and CCL22, factors associated with T_H_2 immune responses, were generally unaffected by β-glucan particle size. Importantly, β-glucan particle size had little effect on mDC activation status; thus, while small β-glucans such as glucan-mp have previously been deemed (from murine studies) to be weak inducers of innate immune responses (14, 15), our data show that this conclusion cannot be generalized to all cytokines and chemokines. We suggest that small β-glucan particles will effectively stimulate human T_H_2 immune responses while not favoring more pro-inflammatory responses, including T_H_17 responses, which require IL-6 and IL-23.

The different cytokine profiles seen with large and small β-glucan particles were shown to be a reflection of the different abilities of these stimuli to induce IL-1β. IL-6, and IL-23 required the large amounts of IL-1β produced in response to curdlan whereas TSLP, while still, as we have previously shown (23, 28), requiring IL-1β for its secretion, was produced in substantial quantities even in the presence of the small amounts of IL-1β generated from glucan-mp-stimulated mDC. This suggests that the threshold concentration of IL-1β required for TSLP production is very low, and much lower than that required for IL-6 and IL-23.

Given the critical difference in the induction of IL-1β by the different sized particles, we investigated the mechanism underlying this and showed that phagocytosis was critical. The small particulate glucan-mp are phagocytosed readily (14), resulting in loss of surface expression of dectin-1. Conversely large particles are not phagocytosed, and surface dectin-1 expression is maintained. By preventing phagocytosis of glucan-mp with CYTD, we inhibited dectin-1 downregulation, completely restored their ability to induce IL-1β, and hence other cytokines, to the same levels as found with curdlan stimulation. We speculate that continued surface expression of dectin-1 allows prolonged stimulation through the receptor, and hence enhanced production of IL-1β. Our data are consistent with Rosas et al. concept that curdlan’s capacity to induce elevated inflammatory cytokine expression is due to “frustrated phagocytosis” (14), and with the idea that this would result in curdlan maintaining dectin-1 expression on the cell surface (15).

In relation to the effects of preventing phagocytosis, we also noted that induction of IL-1β under these conditions was independent of NADPH oxidase activity and most likely ROS production, since CYTD treatment fully restored the ability of mDC from CGD patients to produce IL-1β. In contrast to this, the small amounts of IL-1β induced by glucan-mp from healthy donor mDC were completely absent when mDC from CGD patients were tested suggesting an alternate mechanism of IL-1β production that differs from sustained surface dectin-1 signaling. Thus, the attenuated stimulation through dectin-1, which occurs when small particle β-glucans are used requires the presence of ROS for any IL-1β production. It has previously been shown that both curdlan- and glucan-mp-induced IL-1β requires activation of caspase-1 and caspase-8, to allow pro-IL-1β cleavage by the inflammasome (11, 23). However, with regard to priming of the IL-1β response, we clearly showed that glucan-mp resulted in much lower levels of pro-IL-1β mRNA and protein expression. Given the critical role of IL-1β in antifungal immunity (3, 12, 16), the regulation of its production as determined by β-glucan size likely provides a key molecular checkpoint in eliciting appropriate cytokine secretion from mDC in response to dectin-1 activation.

These findings give insights into the mechanisms DC utilize to orchestrate an effective immune response to fungi. Phagocytosis is an effective cellular mechanism for the removal of foreign organisms; therefore, it may be advantageous for cells, which are capable of phagocytosing fungi, thereby controlling the infection, to limit their production of inflammatory cytokines such as IL-1β that might induce subsequent tissue damage. Pathogenic fungal species such as *C. albicans* exist as both colonizing yeast and invasive hyphae and structural differences exist in β-glucans derived from these differing forms—hyphae exhibit a cyclical β-glucan structure while yeast β-glucans are linear (17). Importantly, β-glucan derived from hyphal *C. albicans* stimulates significantly more IL-1β from DC compared to DC stimulated with β-glucan from yeast (17). Likewise, it has been reported that murine macrophages phagocytose hyphal *C. albicans* more slowly than a strain of *C. albicans*, which is maintained in a yeast form, and prevented from forming hyphae; under these circumstances, the macrophages exhibit frustrated phagocytosis (10).

Our data clearly showed that NADPH oxidase activity and ROS were not required for IL-1β production when phagocytosis was prevented. Previously, de Luca et al. also reported that ROS were not required for induction of IL-1β by *C. albicans* or LPS-primed macrophages (21), but they did not state what form of *C. albicans* was used in these experiments. Our data would be consistent with their using hyphal *C. albicans* and hence induction of frustrated phagocytosis and induction of IL-1β independent of ROS.

There are significant *in vivo* implications of our findings. Dectin-1-stimulated DC potently drive the differentiation of naïve CD4^+^ T cells to T_H_1- and T_H_17-cells required for protective antifungal immunity, and IL-1β, IL-6, and IL-23 are important in generating these T cell subsets. Our data show that naïve CD4^+^ T cells produce higher amounts of IL-17 and IFNγ when stimulated in conditioned media derived from curdlan-stimulated mDC compared to those stimulated with glucan-mp. Therefore, when DC (or macrophages) are confronted with non-invasive yeast, they can clear the infection by rapid phagocytosis, especially macrophages, and limited production of IL-1β, and hence in the case of mDC, which respond to autocrine and macrophage-derived IL-1β IL-6 and IL-23. Conversely, when confronted with invasive hyphal fungi, phagocytosis will be frustrated and an enhanced cytokine response produced with elevated macrophage and mDC production of IL-1β and hence elevated mDC production of IL-6 and IL-23. This in turn will be a critical factor in the stimulation of the T_H_1 and T_H_17 adaptive immune responses needed to clear this more threatening infection.

## Materials and Methods

### Ethics Statement

Human blood was sourced from apheresis cones derived from healthy donors (Addenbrooke’s Hospital, Cambridge) and age- and sex-matched CGD patients (Royal Free Hospital, London). Appropriate consent to use blood-derived cells for research was obtained. Ethics Reference Number: 04/Q0501/119.

### β-Glucan Isolation and Characterization

β-Glucan was isolated from *S. cerevisiae* using a modification of the method of Williams et al. as described by Mueller and colleagues (29, 30). Specifically, phosphoric acid was used instead of hydrochloric acid in the protic acid digestion step. This approach results in a highly pure glucan, but with less degradation of the glucan polymer structure. The glucan was characterized by 1H-NMR as described by Lowman et al. (17). The glucan (~20 mg) was dissolved in 700 µl of deuterated DMSO*_d6_*. Approximately 50 µl of TFA was added to shift the water peak away from the carbohydrate spectral region. The 1H-NMR spectra were obtained at 80°C. *S. cerevisiae* glucan was determined to be >95% pure based on the 1D 1H-NMR and comparison to an ultrapure (>97%) glucan reference standard produced in the Williams laboratory.

### Cell Isolation and Generation of mDCs

Human blood was sourced from healthy donors (Addenbrooke’s Hospital, Cambridge) and age, sex-matched CGD patients (Royal Free Hospital, London). Human mDCs were generated from CD14^+^ monocytes isolated from PBMC by magnetic bead separation (Miltenyi) and were differentiated by culturing for 6 days in RPMI1640 (Lonza) supplemented 5% FCS (Biosera), 20 ng/ml GM-CSF (Life Technologies) and 4 ng/ml IL-4 (BD Biosciences) as described previously (23, 31, 32). Naïve human CD4^+^ T cells were isolated by magnetic bead separation (Miltenyi).

### Stimulation of mDC

Monocyte-derived dendritic cells were stimulated with 50 µg/ml of curdlan (Wako), curdlan microparticles (curdlan-mp) [generated as described previously (14)], and β-1,3 glucan microparticles (glucan-mp) [generated as described above (29, 30)]. mDCs were also stimulated with β-glucans and 2 µM CYTD (Sigma) and/or 10 ng/ml IL-1β (Miltenyi) simultaneously. Cell cultures were incubated for predefined time periods as highlighted in figure legends.

### Differentiation of Naïve CD4^+^ T Cells

Naïve CD4^+^ T cells were stimulated with anti-CD3/anti-CD28 activation beads (1:1 ratio) (ThermoFisher) and 20 IU of IL-2 (Becton Dickinson) for 7 days in the presence of supernatants derived from mDC prestimulated with either 50 µg/ml of curdlan or glucan-mp. CD4^+^ T cell culture supernatants were subsequently harvested to evaluate IL-17 and IFNγ secretion.

### Cytokine Analysis by Enzyme-Linked Immunosorbent Assay (ELISA)

Enzyme-linked immunosorbent assays were performed using appropriate optimized kits for IL-1β, IL-6, IL-23, IL-17, IFNγ (eBioscience), TSLP, and CCL22 (R&D) according to the manufacturers’ instructions. Briefly, plates were precoated with capture antibody overnight and washed five times with wash buffer composed of 1× PBS supplemented with 0.05% Tween 20 (Sigma). This wash protocol was repeated between each protocol step. ELISA plates were then blocked followed by addition of experimental supernatants or standards. Plates were then treated with biotinylated detection antibodies followed by avidin-horseradish peroxidase. 3,3 ′,5,5′-tetramethylbenzidine substrate (eBioscience) was added to each well, with the reaction developed for 10–15 min in the dark. ELISA plates were read at a wavelength of 450 nm with cytokine concentrations calculated using the standard curve values.

### RNA Isolation and Quantitative Real-time Polymerase Chain Reaction (qRT-PCR)

Total RNA was isolated using commercial RNAeasy kits (Bioline) according to the manufacturer’s instructions. mDCs were initially stimulated for 4, 8, or 12 h, supernatants were removed, and cells lysed in lysis buffer. Briefly, lysates were homogenized, placed into spin columns, washed, and centrifuged with commercial diluents and RNA was eluted from the spin columns. qRT-PCR was carried out using TaqMan Gene Expression Assays (Applied Biosystems) for IL-1β, IL-6, IL-23, TSLP, and HPRT. RNA was added to master mix consisting of commercial gene specific probe and primer sets (Applied Biosystems), TaqMan^®^ Universal PCR Master Mix, reverse transcriptase and RNase free H_2_O. Taqman reactions were run for 40 cycles on a predefined temperature cycling program. Gene expression for cytokines were normalized to expression of the house-keeping gene, HPRT, and calculated as either relative expression (2^−ΔCt^) from unstimulated control cells.

### Cell Lysis, Sodium Dodecyl Sulfate (SDS)-Polyacrylamide Gel Electrophoresis (PAGE), and Immunoblotting

Monocyte-derived dendritic cells were lysed in cytoplasmic lysis buffer [10 mM HEPES, 50 mM NaCl, 0.5 M Sucrose, 0.1 mM EDTA, 0.5% v/v Triton X-100, 10 mM Tetrasodium pyrophosphate, 17.5 mM β-glycerophosphate (all from Sigma) and one complete mini protease inhibitor cocktail tablet (Roche)]. Cellular lysates were scraped, and frozen at −20°C overnight before thawing to aid cell lysis. Lysates were centrifuged at 16,000 *g* at 4°C and the cytoplasmic supernatant was separated from the nuclear pellet. Total protein was quantified by Bradford Assay (Thermo). SDS-PAGE was used to resolve proteins. Equivalent concentrations of protein lysate were mixed with 6× loading buffer (10% w/v SDS, 0.3 M Tris–HCl, 25% v/v β-Mercaptoethanol) and glycerol (all from Sigma) and boiled for 10 min. Samples and molecular weight reference proteins were loaded on to precast gradient (4–20%) acrylamide gels (BioRad) and run for 90 min at constant amps (30 mA). Resolved proteins were detected after transfer to nitrocellulose membranes by immunoblot. Membranes were blocked in 5% milk (w/v) (Marvel) and washed three times with wash buffer composed of 1× TBS supplemented with 0.1% Tween 20 between each protocol step. Membranes were then probed with primary antibodies to pro-IL-1β (R&D, AB-201-AB), HIF-1α (Novus, NB100-449), and β-actin (Abcam) overnight, and then incubated in HRP-conjugated secondary antibodies. Protein was detected with ECL (PerkinElmer) and visualized using GBox (Syngene). Protein expression was analyzed relative to β-actin control.

### Flow Cytometry

Flow cytometry was used to determine mDC activation status, dectin-1 expression, and ROS production. For mDC activation status and dectin-1 expression, mDC were resuspended in FACS buffer consisting of PBS supplemented with 0.1% BSA, 2 mM EDTA, and 0.01% Sodium Azide (All Sigma) after stimulation. ROS were detected by incubating mDC with CellRox Green (Thermo) fluorescence dye during stimulations as per manufacturer’s instructions; followed by harvesting into FACS buffer. mDC were then treated with 10% mouse serum (Sigma) for 30 min on ice to block non-specific binding of antibodies to Fc receptors. mDCs were then stained for 30 min with anti-CD11c (Becton Dickinson, Clone B-ly6), anti-CD86 (Biolegend Clone IT2.2), and anti-HLA-DR (Becton Dickinson, Clone 946.6) to examine mDC activation status and anti-dectin-1 (R&D Systems, Clone 259931) to determine dectin-1 downregulation. Cells were then washed, centrifuged at 300 *g* for 5 min, and re-suspended in FACS buffer for analysis. mDC expression of CD11c, CD86, HLA-DR, dectin-1, and ROS was determined by mean fluorescence intensity and acquired using a flow cytometer (FACSFortessa, Becton Dickinson).

### Data Analysis

Cumulative data displayed as mean ± SEM. Data were analyzed using the Prism statistical package (GraphPad) with statistical analysis completed using one-way ANOVA with Bonferroni post-tests (ns = not significant, ****p = 0.001,***p = 0.01, **p = 0.05, *p = 0.1). Flow cytometry data were analyzed using FlowJo 10 software (Treestar).

## Ethics Statement

Human blood was sourced from apheresis cones derived from healthy donors (Addenbrooke’s Hospital, Cambridge) and age- and sex-matched CGD patients (Royal Free Hospital, London). Appropriate consent to use blood-derived cells for research was obtained. Ethics Reference Number: 04/Q0501/119.

## Author Contributions

ME: designed, performed, and analyzed all experimental data and drafted the manuscript. SW: key to experimental design, data interpretation, and reviewed manuscript. RC: facilitated access to patient blood. Aided data interpretation and reviewed manuscript. DW: facilitated access to β-glucan agonists. Aided experimental design, data interpretation, and reviewed manuscript. JSHG: key to experimental design, data interpretation, and reviewed manuscript. JG: key to experimental design, data interpretation, and reviewed manuscript.

## Conflict of Interest Statement

The authors declare that the research was conducted in the absence of any commercial or financial relationships that could be construed as a potential conflict of interest.
