# Supplementary material for: β-Glucan Size Controls Dectin-1-Mediated Immune Responses in Human Dendritic Cells by Regulating IL-1β Production
Source: Front Immunol. 2017 Jul 7;8:791. doi: 10.3389/fimmu.2017.00791 (PMC5500631; doi:10.3389/fimmu.2017.00791)

IMAGE 1

Comparison of the 1D Proton NMR Spectra of the carbohydrate regions plotted from 2.8 to 5.3 ppm of (**A**) *Saccharomyces cerevisiae* glucan and (**B**) Curdlan

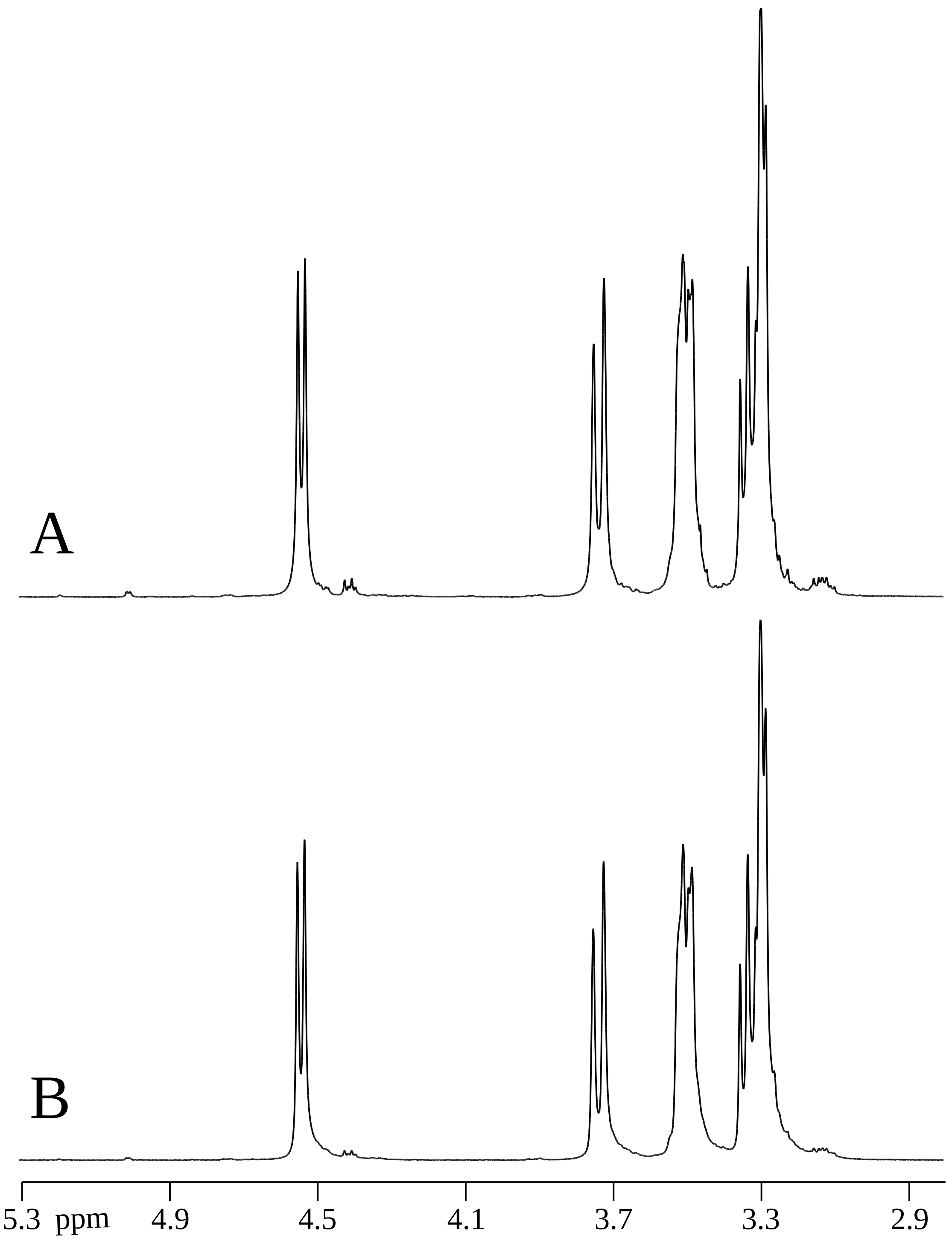

Supplement: Supplementary file 1 [file Image_1.PDF]
